# RNA interference mediated knockdown of *Brugia malayi* UDP-Galactopyranose mutase severely affects parasite viability, embryogenesis and in vivo development of infective larvae

**DOI:** 10.1186/s13071-017-1967-1

**Published:** 2017-01-19

**Authors:** Sweta Misra, Jyoti Gupta, Shailja Misra-Bhattacharya

**Affiliations:** 1 Academy of Scientific and Innovative Research (AcSIR), Anusandhan Bhawan, New Delhi, India; 2 Division of Parasitology CSIR-Central Drug Research Institute, BS 10/1, Sector 10, Jankipuram Extension, Sitapur Road, Lucknow, 226031 UP India

**Keywords:** UDP-Galactopyranose mutase, *Brugia malayi*, Lymphatic filariasis, RNAi, Drug target, siRNA, Embryogenesis

## Abstract

**Background:**

Galactofuranose is an essential cell surface component present in bacteria, fungi and several nematodes such as *Caenorhabditis* spp., *Brugia* spp., *Onchocerca* spp. and *Strongyloides* spp. This sugar maintains the integrity of parasite surface and is essential for virulence. UDP-Galactopyranose mutase (*bmugm*) plays a key role in Gal*f* biosynthesis by catalyzing conversion of UDP-Galactopyranose into UDP-galactofuranose and knockout studies of the gene in *Leishmania major*, *Mycobacterium* and *Aspergillus fumigatus* displayed attenuated virulence while RNA interference study in *C. elegans* exhibited detrimental effects. Presence of UGM in several prokaryotic and eukaryotic microbial pathogens and its absence in higher eukaryotes renders it an attractive drug target. In the present study, RNA interference studies have been carried out to validate *bmugm* as an antifilarial drug target.

**Methods:**

RNA interference studies using two different sequences of siRNAs targeting *bmugm* were carried out. The in vitro gene silencing of adult *B. malayi* parasites was undertaken to observe the effects on parasites. Infective larvae were also exposed to siRNAs and their in vivo development in jirds was observed.

**Results:**

The in vitro gene silencing induced by siRNA1 and 2 individually as well as together knocked down the *bmugm gene* expression causing impaired viability of the exposed worms along with extremely reduced motility, abridged microfilarial release and adversely effected embryogenesis. The combinatorial in vitro gene silencing revealed marginally better results than both the siRNAs individually. Thus, infective larvae were treated with siRNA combination which showed downregulation of *bmugm* mRNA expression resulting into sluggish larval movements and/or death. The siRNA-treated actively motile larvae when inoculated intraperitoneally into jirds demonstrated highly reduced transformation of these larvae into adult worms with detrimental effects on embryogenesis. The effects of gene silencing were long-lasting as the adult worms developed from siRNA-treated larvae showed noticeable knockdown in the target gene expression.

**Conclusions:**

The validation studies undertaken here conclude that *bmugm* is essential for the proper development and survival of the parasite and support its candidature as an antifilarial drug target.

**Electronic supplementary material:**

The online version of this article (doi:10.1186/s13071-017-1967-1) contains supplementary material, which is available to authorized users.

## Background

Lymphatic filariasis (LF) is a vector-borne, painful and disfiguring parasitic disease caused by *Wuchereria bancrofti*, *Brugia malayi* and *Brugia timori*, leading to severe socio-economic consequences. Around 1.23 billion inhabitants in 58 countries are at risk of acquiring infection and therefore urgently require large scale preventive measures [1]. The current standard drugs ivermectin and diethylcarbamazine in combination with albendazole are largely microfilaricidal, moreover they need to be administered in a single dose, annually for several years for proper interruption of the microfilarial transmission. The development of resistance against albendazole and ivermectin has been well established [2], which highlights the urgent need of excavating novel drug targets to discover adulticidal/embryostatic/microfilaricidal agents. The reverse genetic approach may be of help in this regard and RNA interference (RNAi) provides a valuable modern drug discovery platform for identifying gene(s) playing vital role in parasite metabolism by inhibiting/silencing their expression.

Galactofuranose (Gal*f*), a five member ring form of galactose, is an essential component of cell wall, cell surface glycolipids and glycoproteins. It is widely distributed among several nematodes such as the free-living *Caenorhabditis elegans* and parasitic *Brugia* spp., *Onchocerca* spp. and *Strongyloides* spp., as well as protozoans and prokaryotes [3]. In mammals, this sugar is exclusively found in hexopyranosyl form (Gal*p*). The ubiquitous presence of Gal*f* in non-mammalian species is remarkable and its expression in many pathogens suggests that it may be an essential element for survival. Absence of Gal*f* in these organisms often results in morphological abnormalities and an impaired cell wall function. Gal*f*-deficient mutants have been found to be hypersensitive to drugs, exhibited constitutive osmotic stress phenotype or had an attenuated virulence [4]. Thus, apart from maintaining the parasite surface integrity, Gal*f* also appears to be essential for their virulence. Gal*f*-biosynthetic pathways have attracted quite a lot of interest as targets for drug development against microbial infections. The flavoenzyme, UDP-Galactopyranose mutase (UGM) plays a key role in Gal*f* biosynthetic pathway by catalyzing the conversion of UDP-Galactopyranose (UDP-Gal*p*) into UDP-galactofuranose (UDP-Gal*f*) [5]. Eukaryotic UGMs share nearly 25–30% similarity with bacterial UGM and most of the substrate or FAD binding residues have remained conserved [6]. UGM knockouts of *Leishmania major* and mutants of *Aspergillus fumigatus* displayed decreased/attenuated virulence [7, 8] while *C. elegans* mutants exhibited larval lethality and severe embryonic phenotypic deformities indicative of defective surface coat synthesis [9]. Genome-wide RNAi screens in *C. elegans* suggested that downregulation of *glf-1* gene (an orthologue of *B. malayi* UGM) is detrimental [10–14]. Presence of UGM in several prokaryotic and eukaryotic microbial pathogens and its absence in higher eukaryotes renders it an attractive drug target. An *in silico* study has also confirmed its relevance as a candidate antifilarial drug target [15]. Thus, in the present study, in vitro and in vivo validation of *B. malayi* UGM as putative antifilarial drug target has been carried out by siRNA mediated gene silencing to understand the biological function of the enzyme in human lymphatic filarial parasite, *B. malayi*.

## Methods

### Customized synthesis of siRNA

siRNAs (small interfering ribonucleic acids) were custom synthesized by Dharmacon (USA) to target *B. malayi* UGM (*bmugm*) (NCBI: FJ860969.1). Two sequences of siRNAs labeled with FAM (6-carboxyfluorescein) showing highest specificity ranking were procured in the lyophilized form. Stock solutions (100 μM) were prepared and stored at -80 °C.

Custom synthesized stable scrambled siRNA sequence (negative control) tagged with FAM not bearing any sequence similarities with that of human, rat or mouse and not targeting any gene of *B. malayi* was used to observe off target effects, if any. This scrambled siRNA was prior tested in cell based screening where it showed no effect on cell viability, morphology or proliferation. All the siRNAs were sistable which means that they were modified for nuclease resistance to enhance their stability even in nuclease-rich environments. Sequences of target based and scrambled siRNAs are given in Table 1.

**Table 1:** The siRNA sequences used for RNAi studies

	siRNA		Sequence (5'–3')
Targeting *bmugm*	siRNA1	sense	CAGUAUUAGUGAAGAAGAAUU
		anti-sense	UUGUCAUAAUCACUUCUUCUU
	siRNA2	sense	GGAAAUAUGAGGUAUCAAAUU
		anti-sense	UUCCUUUAUACUCCAUAGUUU
Non-targeting scrambled (negative control)		sense	UAGCGACUAAACACAUCAAUU
		anti-sense	UUAUCGCUGAUUUGUGUAGUUUU

### Parasite isolation from infected jirds

Adult *B. malayi* parasites were recovered from jirds (*Meriones unguiculatus*) within 3–5 months of intraperitoneal inoculation of infective larvae (L3) of *B. malayi*. L3 were recovered from infected *Aedes aegypti* mosquitοes washed in culture medium repeatedly as described earlier [16]. The worms were retrieved by washing the peritoneal cavity of jird. The recovered worms were washed in culture medium RPMI-1640 containing 2 mM L-glutamine, 25 mM HEPES, 100 U/ml penicillin, 100 mg/ml streptomycin and 2.5 mg/ml amphοtericin B. Worms were placed in a 48-well plate keeping 1 wοrm in 1 ml of culture medium per well and plate was kept at 37 °C under 5% CO_2_ for at least 2 h to select undamaged, healthy and highly motile worms (male and female) for siRNA treatment.

### In vitro siRNA treatment of worms by soaking method

The healthy male and female adult worms were used for in vitro gene silencing studies by soaking methοd as described earlier with some modifications [17]. Five male and five female adult worms were placed in Midi GeBaflex tubes with molecular weight cut off (MWCO) of 6–8 kDa (GeBa, Israel) containing 800 μl οf culture medium fortified with 8 U RNase OUT, 1 mM spermidine and 5 μM of target siRNA. The effective concentration of siRNA has been optimized earlier in our lab targeted against other *B. malayi* genes [17, 18]. The tubes with scrambled siRNA and no siRNA instead of target siRNA served as their respective negative and medium controls. FAM labelled stable siRNAs siRNA1 and siRNA2 targeting *bmugm* mRNA were used alone as well as in combination for analyzing their effect on worms at different time points. Five tubes (medium + worms) were kept in a 250 ml capacity screw capped glass bottle (Schott) containing 200 ml of RPMI medium preheated to 37 °C and enough care was taken in order to avoid any contamination, stress and damage to the parasites. Of these five tubes, tube 1, 2, 3, 4, 5 contained siRNA1 (5 μM), siRNA2 (5 μM), siRNA1 *+* siRNA2 at 2.5 μM each, scrambled siRNA (negative control) (5 μM) and no siRNA (medium control), respectively. Five such sets of bottles were prepared for retrieving tubes at 24, 48, 72 h and the last two for observing the post-silencing effects. Tubes from bottle 1 were retrieved after 24 h of incubation and adult worms were transferred to fresh medium in separate petridishes. The medium left (800 μl) in these five tubes was transferred to separate eppendorfs, centrifuged at 800 × *g* for 2 min to pellet microfilariae (mf) released in vitro by female worms. The mf pellets were suspended in 50 μl fresh medium and microscopically examined at different time points during (24, 48, 72 h), post-gene silencing (96, 120 h) for enumeration. Ten microliters of mf suspension was spread into thin smear on a glass slide and stained with Giemsa for observing the phenotypic changes, if any. The images were captured by colour digital camera (Nikon, Tokyo, Japan).

Penetration of siRNA into the treated worm was affirmed by visualization under the fluorescent microscope (FLoid Cell Imaging Station; Life technologies, CA, USA) at 520 nm emission. However, size/stage-matched parasites were also captured for autofluorescence if any. Two female worms and three male worms from each tube were frozen in Trizol for nucleic acid preparation to measure target gene mRNA expression employing real-time quantitative RT-PCR (qRT-PCR). Further, two male and two female worms were transferred to fresh culture medium at 37 °C for 30 min to assess their motility and percentage inhibition in motility, if any. The viability of these worms was evaluated by MTT [3-(4, 5 dimethylthiazol-2-yl)-2, 5 diphenyltetrazolium bromide] reduction assay [19, 20]. The remaining one female was teased and microscopically examined for intrauterine contents to determine the effect of gene silencing on embryogenesis and other developing progenies. Worms kept in the tubes in two other bottles were also processed in the same way after retrieving parasites at 48 and 72 h respectively. Worms from the last two bottles were examined for post-silencing effects beyond 72 h after transferring treated worms to siRNA-free fresh medium and further incubating them for another 24 and 48 h respectively. These worms were examined for motility, viability and intra uterine contents and remaining parasites were frozen in Trizol for further qRT-PCR. Mf release was also quantified as mentioned earlier. The data stated in the present study are the mean ± standard deviation (SD) of the three experiments (in each experiment any siRNA type or controls were kept in duplicates for each time point) carried out with the same number of worms under identical conditions.

### Effect on motility and viability of *B. malayi* adult worms and microfilariae

The adult worms (both sexes) from each group of target siRNA soaked, scrambled negative and medium control were analyzed for their motility post-transfer to fresh media. Reduction in motility was analyzed microscopically and % motility reduction was scored as follows: 5, 1–25% reduction; 4, 26–49% reduction; 3, 50–74% reduction; 2, 75–99% reduction; and D (dead), 100% reduction. MTT utilizes mitochondrial enzyme to reduce yellow dye into blue formazon, which is solubilised in DMSO and read spectrophotometrically in a multiplate reader (Tecan, Infinite M-200, Mannedorf, Switzerland) at 540 nm indicating viability of worms. Reduction in motility and inhibition in MTT reduction by treated worm in comparison to those of respective controls indicated the effect of gene silencing on *B. malayi*.

### *bmugm* mRNA expression analysis by qRT-PCR

The downregulation in the mRNA expression levels of target specific transcript in the treated and control worms was quantified using qRT-PCR. *β-tubulin* gene (Bm-tub-1) was used as an endogenous control. The target and housekeeping gene-specific primers were designed (Eurogentec, Seraing, Belgium) for qRT-PCR (Table 2).

**Table 2:** The primers sequences used for real time RT-PCR analysis

Real time qPCR primers		Sequence (5'–3')
*bmugm*	sense	CACCTGGTGGATTAAGTCGAACAG
	anti-sense	AGATGCAAGCCTTTCTCGTTGAAC
*β-tubulin*	sense	ACTTGGTGTCCGAATATC
	anti-sense	ACTCTTCCTGTTCAATGTAT

The treated and control worms frozen in Trizol at different time points (as mentioned above) were used for RNA extraction by Trizol method [21] followed by DNase treatment and cDNA synthesis using Super Script III first strand cDNA synthesis kit (Invitrogen, CA, USA) containing oligo (dT) 20 primers. The cDNA was amplified by qRT-PCR using the real-time PCR primers and SYBER GREEN premix on Roche applied System (Indianapolis, USA). PCR amplification was carried out at 95 °C for 5 min, followed by 40 cycles of 95 °C for 20 s, 56 °C for 15 s, and 72 °C for 30 s using target specific and endogenous control gene primers. The relative fold change in mRNA expression was determined by comparative ΔCT method [22].

To further support the above qRT-PCR findings a separate set of experiments were conducted to check the changes in *B. malayi* UGM protein (Bm_UGM) levels in treated and untreated worms. Polyclonal antibodies were raised against the protein in BALB/c mice (6–8 week old) and anti-Bm_UGM serum was collected from the blood as mentioned earlier in [23]. The worms (3 males + 2 females) from each group after treatment with medium alone and siRNAs (scrambled, siRNA1, 2, 1 + 2) at different time points as above were homogenized, sonicated in 300 μl PBS containing protease inhibitor cocktail (Sigma, St Louis, Missouri, USA), protein content quantified by Bradford method and 20 μg of each sample was resolved on 10% SDS-PAGE for Western blotting using anti-Bm_UGM mouse serum as a primary antibody (1:5000 dilutions). The blot was developed as mentioned earlier [23] and percent relative intensity of bands in comparison to medium control was quantified by using open-source imageJ version 1.47 software (NIH, Bethesda, MD, USA).

### In vitro gene silencing in L3 and their in vivo development in jirds

*Brugia malayi* L3 were kept in 48 well plate ~200 L3/ml/well in culture medium fortified with 8 U RNase OUT, 1 mM spermidine at 37 °C in 5% CO_2_ [18]. Larvae were soaked in medium containing target siRNA1 + 2 (2 μM), scrambled siRNA (2 μM) and no siRNA (medium control) in multiple wells for 48 h. Treated as well as control L3 were frozen in Trizol for further quantification of their transcript levels. After washing, the healthy and actively motile larvae from each treated and control groups were inoculated into the peritoneal cavity of 6 week-old susceptible rodent host (male jird) for examining their further in vivo development. Four jirds were inoculated each with ~100 L3/animal recovered from each group of wells. These jirds were euthanized on day 120 post-larval inoculation for observing the development of L3 into sexually mature, fertile adult worms and presence of mf in peritoneal cavity. The live recovered worms were measured for their length and teased in PBS to detect defects in intrauterine development, if any.

### Statistical analysis

The in vitro and in vivo experiments were repeated three times under similar conditions to minimize the errors, if any. *T*-test (for two groups), two-way analysis of variance (ANOVA) were used for analyzing the data. Bonferroni method/Newman-Keuls Multiple Comparison Test was used for making individual comparisons using PRISM 5.0 and STATISTICA 7.0 statistical software wherever applicable. Statistical significance between experimental and control groups was evaluated as *P*-values ≤ 0.05, ≤ 0.01 and ≤ 0.001 which were considered to be significant, highly significant, very highly significant and marked as *, ** and ***, respectively. The probability of each individual comparison was at 100 *df* (degrees of freedom) for *vitro* real time data of adult parasites after ANOVA while, in vivo studies (adulticidal, sterilization and real time) the critical probability for *t*-test at 22 *df* was used.

## Results

### Visualization of siRNA uptake by parasites

**Fig. 1:**
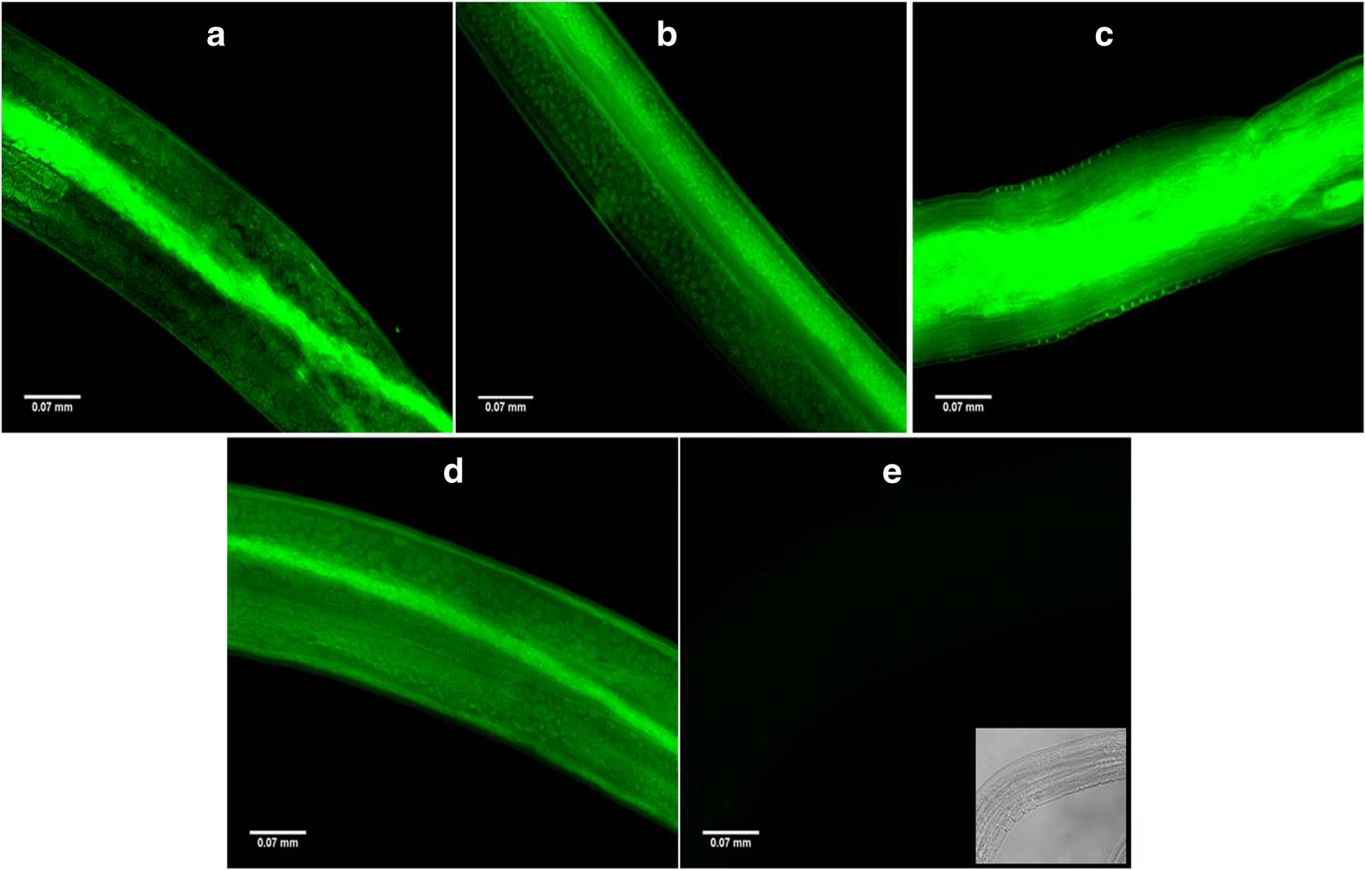
Successful uptake of siRNAs by female *B. malayi* parasites of different groups siRNA1 (**a**), siRNA2 (**b**), siRNA1 + 2 (**c**), scrambled siRNA (negative control) (**d**), and unexposed worm with phase contrast image at right bottom (**e**) after 24 h. The images were taken under FLoid Cell Imaging Station (Life technologies, US) at 520 nm emission

**Fig. 2:**
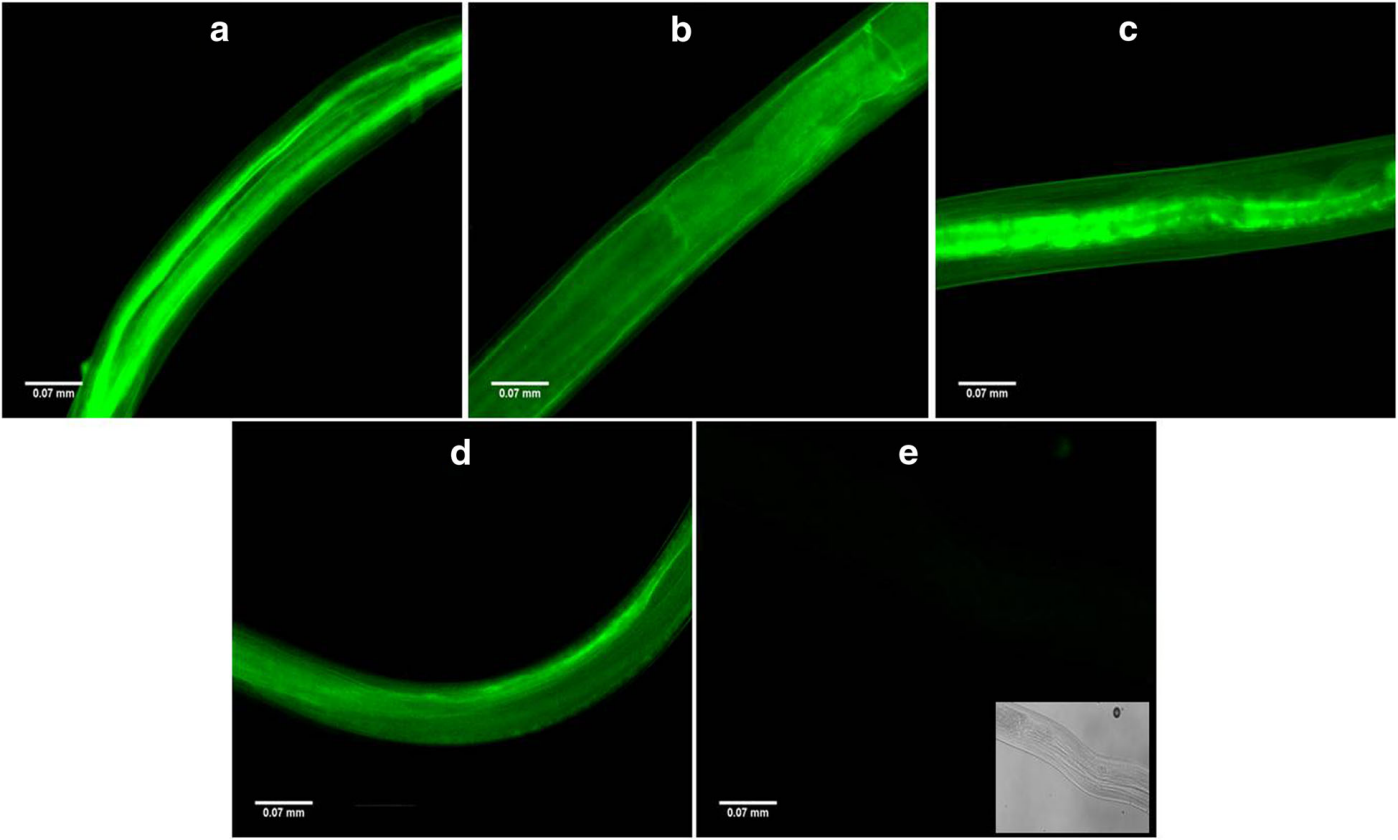
Successful uptake of siRNAs by male *B. malayi* parasites of different groups siRNA1 (**a**), siRNA2 (**b**), siRNA1 + 2 (**c**), scrambled siRNA (negative control) (**d**) and unexposed worm with phase contrast image at right bottom (**e**) after 24 h under the fluorescent microscope. The images were taken under FLoid Cell Imaging Station (Life technologies, US) at 520 nm emission

**Fig. 3:**
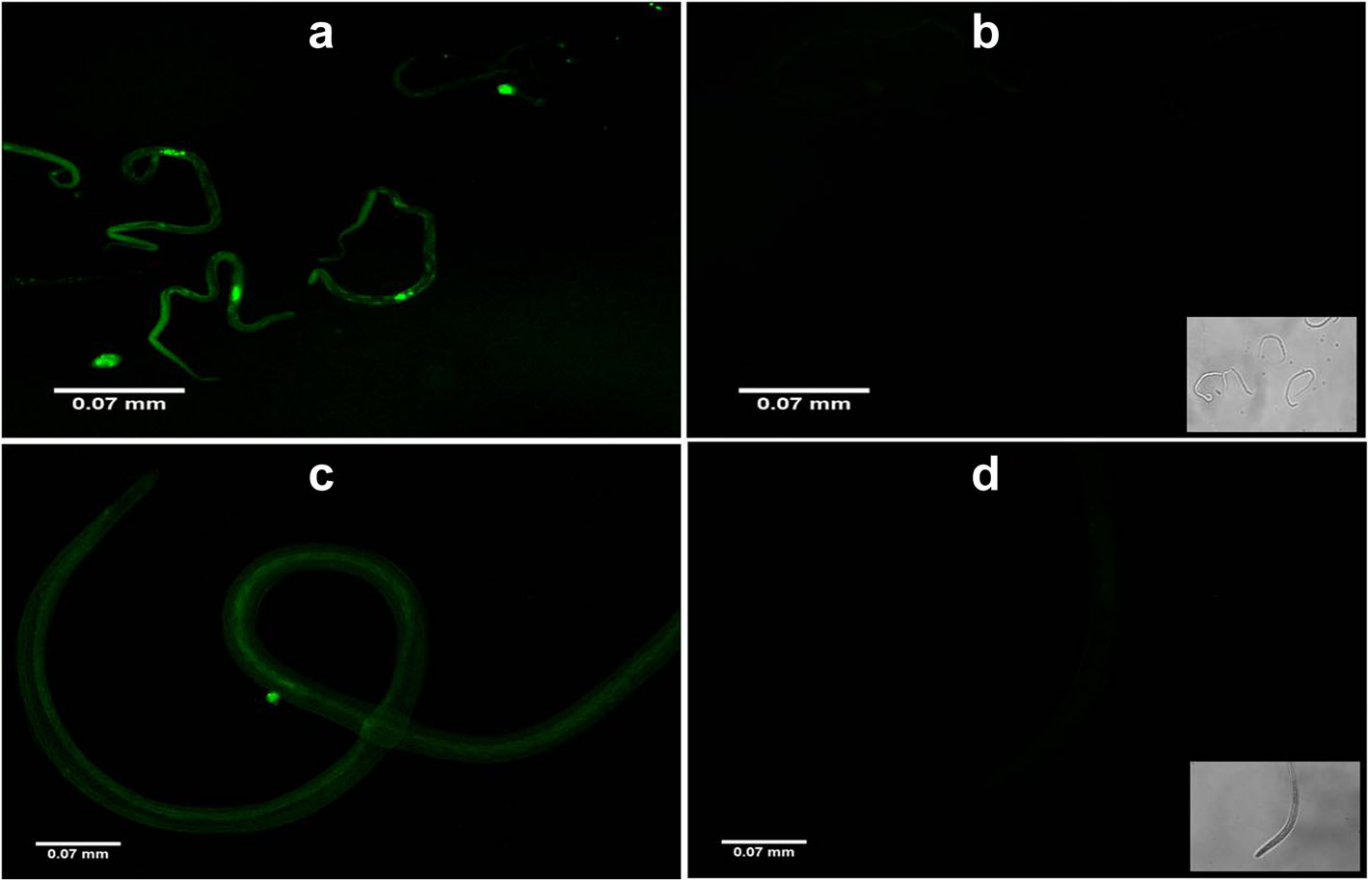
Successful uptake of siRNAs by *B. malayi* microfilariae and infective larvae. The images show successful siRNA uptake by *B. malayi* microfilariae released by siRNA-treated female parasites of different groups (**a**), unexposed control microfilariae (with phase contrast image at right bottom) (**b**), siRNA-treated infective larvae (**c**), and unexposed control infective larvae (with phase contrast image at right bottom) (**d**) after 24 h under the fluorescent microscope. The images were taken under FLoid Cell Imaging Station (Life technologies, US) at 520 nm emission

There was successful uptake of FAM labelled stable off target siRNA (scrambled), siRNA1, siRNA2 and both siRNA1 + 2 by the parasites on soaking them for 24 h which was visible equally throughout the body of females (Fig. 1a-d), males (Fig. 2a-d), mf released by siRNA-treated worms (Fig. 3a) and L3 (siRNA1 + 2 soaked) (Fig. 3b) till the end of exposure. The possibility of autofluorescence was ruled out by capturing the size/stage matched images of unexposed parasites as shown in various figures pertaining to adult female (Fig. 1e), adult male (Fig. 2e), mf (Fig. 3b), L3 (Fig. 3d). Phase contrast images are also included in each figure at right bottom.

### Impaired motility and viability of adult *B. malayi*

Silencing of *bmugm* mediated through siRNA1, siRNA2 and siRNA1 + 2 caused 50–65% inhibition in motility of adult female and 60–70% in male wοrms within 24 h of soaking bringing down their viability between 58 and 70%. The motility of worms decreased tremendously at the end of 72 h (98–99%) and did not revive on transferring the treated worm to siRNA free fresh medium even up to 48 h. The worms were almost paralyzed and only their distal end sometimes showed jerky movement on stirring the medium. The viability and motility of treated worms was reduced by 90–98% between 72 and 120 h (Tables 3 and 4). These effects were more intense when parasites were soaked in a mixture of two target siRNAs.

**Table 3:** Effect of *bmugm* gene silencing on viability of adult *B. malayi* female parasites assessed by motility scoring and MTT reduction assay

Time (h)	Motility score (female worms)^a^					% inhibition in MTT reduction (mean ± SD)			
	MC	SCR	siRNA 1	siRNA 2	siRNA 1 + 2	SCR	siRNA 1	siRNA 2	siRNA 1 + 2
24	5+	5+	2+	2+	2+	5.2 ± 0.40	66.1 ± 5.58**	57.9 ± 0.80**	58.8 ± 9.89**
48	5+	4+	1+	1+	1+	3.7 ± 3.40	86.9 ± 1.70***	83.4 ± 3.67***	87.6 ± 1.9***
72	4+	4+	1+	1+	1+	11.1 ± 1.84	87.3 ± 9.98***	88.6 ± 1.58***	98.1 ± 2.26***
96	4+	4+	1+	1+	1+	11.3 ± 3.17	88.7 ± 2.47***	91.5 ± 1.93***	92.2 ± 2.64***
120	4+	4+	1+	1+	1+	5.3 ± 0.83	87.8 ± 3.9***	87.4 ± 2.8***	90.1 ± 3.73***

*Abbreviations*: *MC* medium control; *MTT* (3-(4, 5 dimethylthiazol-2-yl)-2, 5 diphenyltetrazolium bromide); *SCR* negative control (scrambled siRNA); *SD* standard deviation

**P < 0.01; ***P < 0.001

^a^5, 0% motility reduction; 4, 1–25% reduction; 3, 26–49% reduction; 2, 50–74% reduction; 1, 75–99% reduction; D (death), 100% reduction

**Table 4:** Effect of *bmugm* gene silencing on viability of adult *B. malayi* male parasites was assessed by motility scoring and MTT reduction assay

Time (h)	Motility score (male worms)^a^					% inhibition in MTT reduction (mean ± SD)			
	MC	SCR	siRNA 1	siRNA 2	siRNA 1 + 2	NC	siRNA 1	siRNA 2	siRNA 1 + 2
24	5+	5+	2+	2+	2+	11.6 ± 1.98	69.1 ± 9.93**	69.8 ± 2.90**	69.9 ± 2.13**
48	4+	4+	1+	1+	1+	5.9 ± 2.95	60.7 ± 10.47**	77.2 ± 8.97***	84.9 ± 11.95***
72	4+	4+	1+	1+	1+	7.1 ± 3.68	80.5 ± 9.27***	85.70 ± 8.50***	92.7 ± 0.88***
96	3/4+	3/4+	1+	1+	1+	6.2 ± 2.35	90.8 ± 3.60***	87.3 ± 9.60***	90.6 ± 9.53***
120	3/4+	3/4+	1+	1+	1+	7.9 ± 4.13	85.2 ± 2.75***	83.5 ± 9.85***	90.9 ± 1.83***

*Abbreviations*: *NC* negative control (scrambled siRNA); *MC* medium control, *MTT* (3-(4, 5 dimethylthiazol-2-yl)-2, 5 diphenyltetrazolium bromide); *SCR* negative control (scrambled siRNA); *SD* standard deviation

**P < 0.01; ***P < 0.001

^a^5, 0% motility reduction; 4, 1–25% reduction; 3, 26–49% reduction; 2, 50–74% reduction; 1, 75–99% reduction; D (death), 100% reduction

### Reduced mf release, viability and alteration in phenotype

**Fig. 4:**
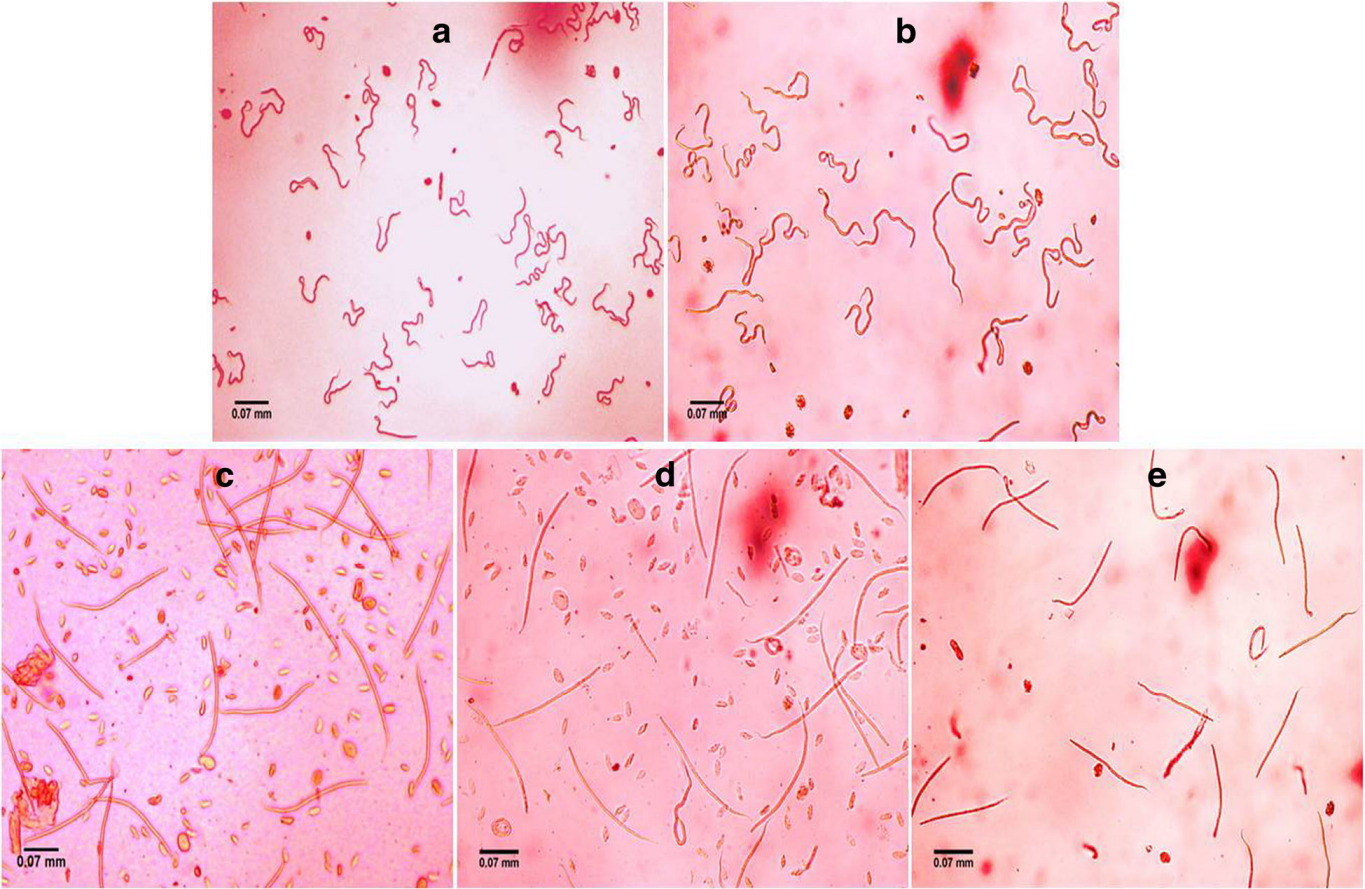
Effect of in vitro gene silencing on the microfilariae released by female *B. malayi* adult worm. **a**, **b** depict the healthy and motile microfilariae of medium control (**a**) and negative control (scrambled siRNA) (**b**), dead and severely sluggish microfilariae released by siRNA1 (**c**), siRNA2 (**d**), siRNA1 + 2-treated female parasites in vitro (**e**)

**Fig. 5:**
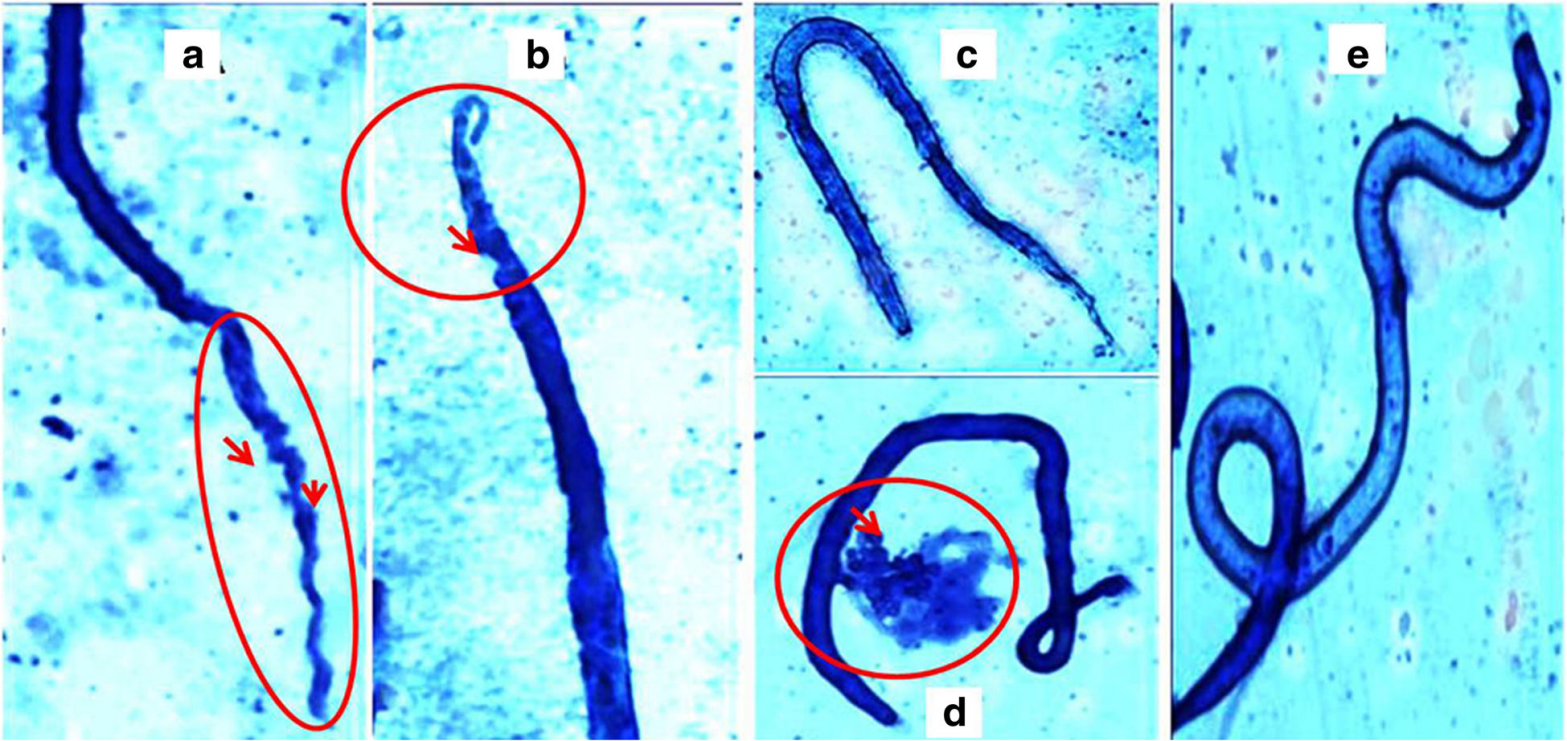
Phenotypic deformities observed in microfilariae. The microfilariae released by the treated *B. malayi* adult worms were stained with Giemsa stain for observing the phenotype. The microfilariae recovered from the siRNA-treated groups showed slight deformities (**a**-**d**) as encircled and marked (*red*) in respective images in comparison to the intact microfilariae released in control groups (**e**)

siRNA1, 2 and in combination significantly reduced the release of mf from female worms in culture. The percent decrease in mf density was 75 ± 2.26 (siRNA1), 73.95 ± 5.59 (siRNA2) and 79.45 ± 2.20 (siRNA1 + 2) within 24 h which went down further to ~84–86% at 72 h and ~90% after further 48 h of transfer to siRNA-free medium (Table 5). As expected, targeting of *bmugm* by two distinct sequences specific siRNAs at any time point proved to be marginally better. No off-target effects were noticed in presence of scrambled siRNA. Mf released in control cultures were healthy, actively motile (Fig. 4a, b) unlike those targeted specifically. Significant reduction (65–75%) in the motility of released mf was observed within 24 h which was exemplified with increased exposure time (>90% after 48 h). Majority of mf released at 72 h were dead (Fig. 4c-e and Table 6) with visible phenotypic deformities (Fig. 5a-d) as compared to intact mf from controls (Fig. 5e).

**Table 5:** Effect of siRNA treatment on microfilaria release by female *B. malayi* adult parasites

Time (h)	% inhibition in microfilaria release (mean ± SD)			
	SCR	siRNA1	siRNA2	siRNA1 + 2
24	4.60 ± 0.85	75.4 ± 2.26***	73.9 ± 5.59***	79.4 ± 2.20***
48	5.3 ± 1.25	79.7 ± 1.84***	80.0 ± 2.97***	81.2 ± 1.96***
72	4.3 ± 0.71	85.3 ± 1.69***	84.7 ± 2.97***	86.3 ± 1.20***
96	3.7 ± 1.56	88.3 ± 0.99***	87.9 ± 2.26***	88.8 ± 1.69***
120	3.6 ± 1.56	89.5 ± 0.71***	89.2 ± 2.26***	90.3 ± 0.99***

*Abbreviations*: *MC* medium control; *SCR* negative control (scrambled siRNA); *SD* standard deviation

***P < 0.001

**Table 6:** Percentage reduction in motility of microfilariae released by *B. malayi* female adult parasites after in vitro RNA interference was analyzed by scoring their motility

Time (h)	Reduction in microfilaria motility^a^				
	MC	SCR	siRNA1	siRNA 2	siRNA1 + 2
24	5+	5+	2+	2+	1+
48	5+	5+	1+	1+	1+
72	5+	5+	1+/D	1+/D	1+/D
96	5+	5+	1+/D	1+/D	1+/D
120	5+	5+	1+/D	1+/D	1+/D

*Abbreviations*: *MC* medium control; *SCR* negative control (scrambled siRNA)

^a^5, 0% motility reduction; 4, 1–25% reduction; 3, 26–49% reduction; 2, 50–74% reduction; 1, 75–99% reduction; D (death), 100% reduction

### Impaired embryogenesis in female worm

**Fig. 6:**
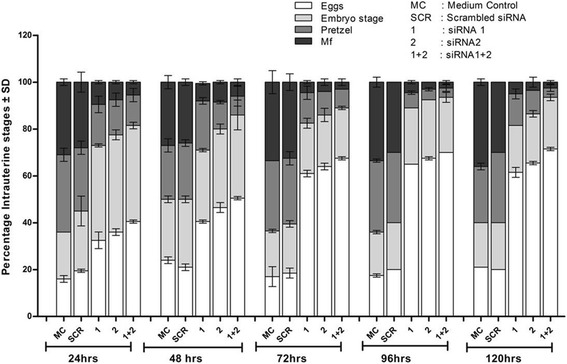
Gene silencing effects on embryogenesis. The effect on adult female worm embryogenesis after treatment with siRNA1, 2, 1 + 2, scrambled and medium was analyzed. The intrauterine progenies from individual female worms of treated and control groups were examined microscopically and are expressed as the relative proportions of progeny at different stages of development. The data represent the percentage ± standard deviation (SD) of eggs, embryonic stages, pretzels and microfilariae

**Fig. 7:**
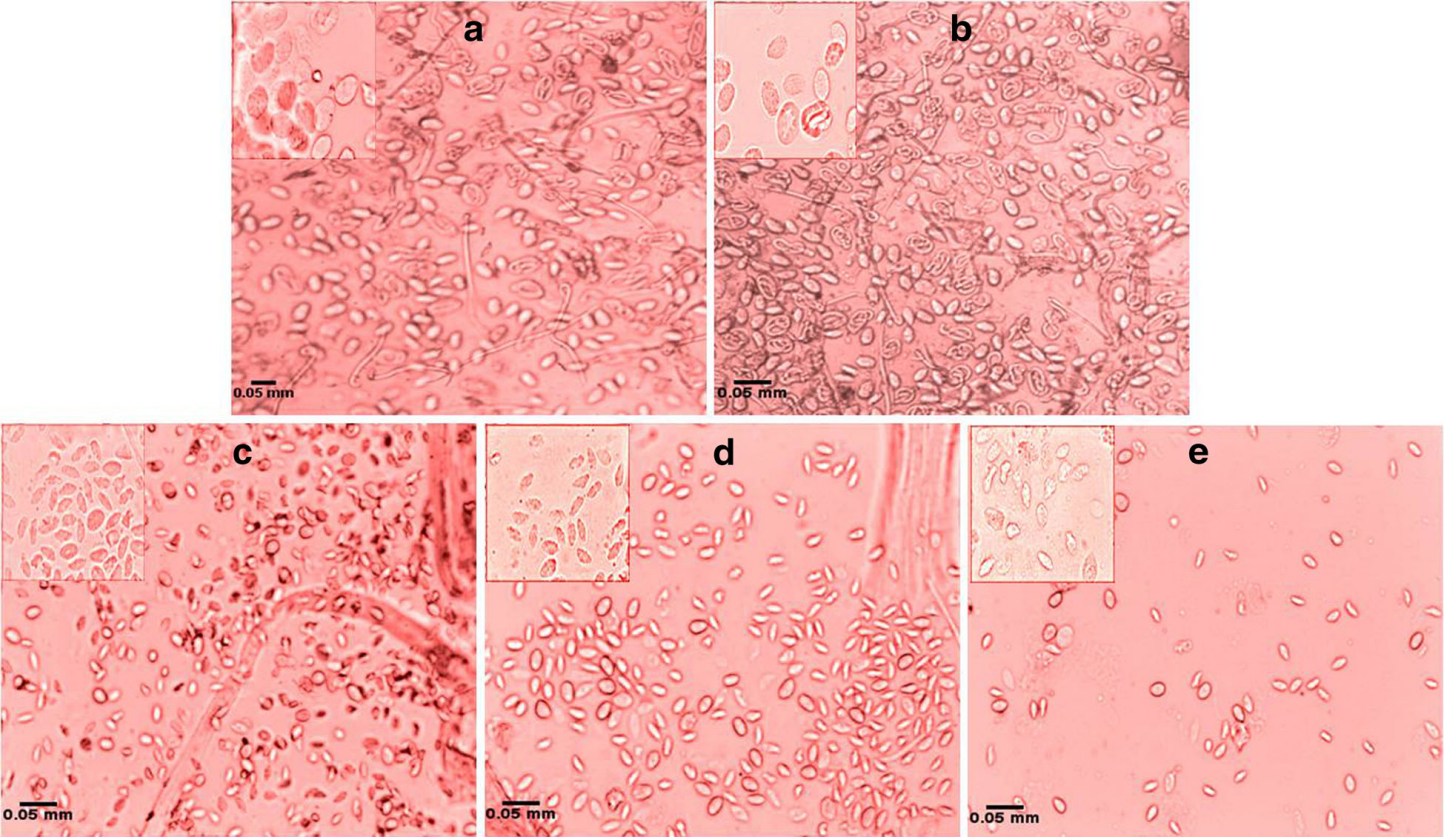
Phenotypic changes observed in the eggs. After treatment of adult female *B. malayi* with siRNA1 (**c**), siRNA2 (**d**), siRNA1 + 2 (**e**) the eggs were degenerated (shrunken) in comparison to medium control (**a**) and negative control (scrambled siRNA) (**b**). The respective zoomed images of eggs depicting their degeneration are shown on the upper left side of each image

An adverse effect was also noticed on intrauterine contents post-*bmugm* gene silencing in female *B. malayi*. All of the stages including eggs, pretzel, advanced embryonic and microfilarial stages showed degeneration with decrease in their proportions. Female teased at 120 h contained 66–71% eggs, 20–23% embryonic stages, 5–15% pretzel stages and merely 2–5% mf which were much less than in control females. In the specifically targeted worms, percentage of early stages increased significantly with concomitant dwindled counts of advanced developing stages (Fig. 6). The eggs from the two control groups were normal (Fig. 7a, b) as opposed to degenerated eggs in the female worms treated with siRNA1 (Fig. 7c), siRNA2 (Fig. 7d) and siRNA1 + 2 (Fig. 7e), these were either granulated and/or shrunken (zoomed view of eggs for showing their condition given on upper left side of each image in Fig. 7).

### Specific gene silencing led to *bmugm* transcript knockdown

**Fig. 8:**
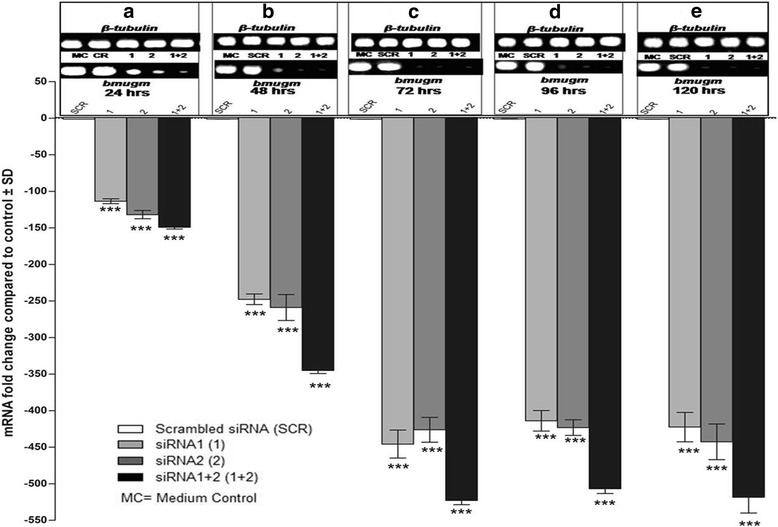
Knockdown in *bmugm* gene expression in adult *B. malayi* female parasites. The relative fold downregulation in mRNA expression of *bmugm* in *B. malayi* adult female worms soaked in siRNA1, 2, 1 + 2 was quantified by real time PCR (qRT-PCR) using comparative ΔCT method at different time interval (24, 48, 72, 96, 120 h), *β-tubulin* was the endogenous control housekeeping gene. The above results depict the mean ± standard deviation (SD) derived from three separate experiments carried out under similar conditions. The gel images depicting the target (*bmugm*) and endogenous control (*β-tubulin*) gene expression status at different time points are shown on top (**a**-**e**). ***P < 0.001 (very highly significant)

**Fig. 9:**
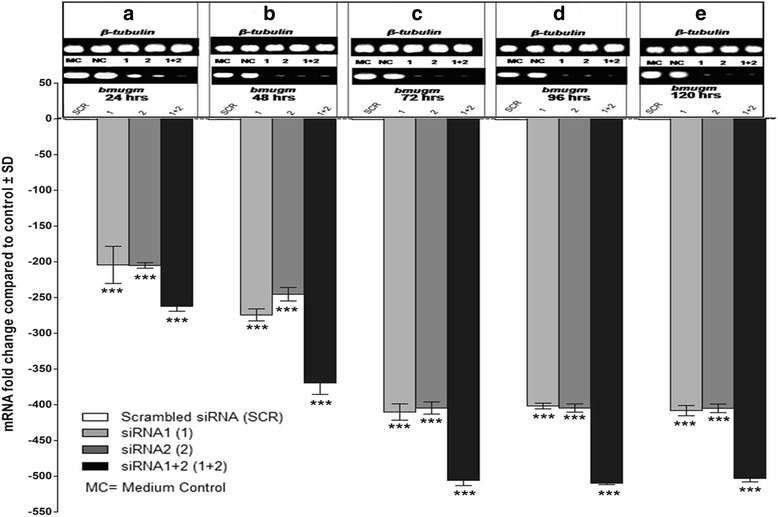
Knockdown in *bmugm* gene expression in adult *B. malayi* male parasites. The relative fold downregulation in mRNA expression of *bmugm* in *B. malayi* adult male worms soaked in siRNA1, 2, 1 + 2 was quantified by real time PCR (qRT-PCR) using comparative ΔCT method at different time interval (24, 48, 72, 96, 120 h), *β-tubulin* was the endogenous control housekeeping gene. The above results depict the mean ± standard deviation (SD) derived from three separate experiments carried out under similar conditions. The gel images depicting the target (*bmugm*) and endogenous control (*β-tubulin*) gene expression status at different time points are shown on top (**a**-**e**). ***P < 0.001 (very highly significant)

The statistical analysis (two way ANOVA followed by pairwise comparisons) showed significant changes among groups (*F*_(3,100)_ = 6761, *P* < 0.001), at different time points (*F*_(4,100)_ = 1998, *P* < 0.001). Profound downregulation in the *bmugm* gene expression was noticeable following in vitro siRNA treatment to adult *B. malayi*. In female worms, mean *bmugm* transcript level was cut down by approximately 113-fold (*t*_(100)_ = 16.05, *P* < 0.001), 131-fold (*t*_(100)_ = 18.67, *P* < 0.001) and 149-fold (*t*_(100)_ = 21.14, *P* < 0.001) within 24 h in presence of siRNA1, siRNA2 and siRNA1 + 2, respectively, which reduced further to 445-fold (*t*_(100)_ = 63.51, *P* < 0.001), 426-fold (*t*_(100)_ = 60.72, *P* < 0.001) and 523-fold (*t*_(100)_ = 74.54, *P* < 0.001) over medium control within 72 h (Fig. 8a-f). The transcript levels were normalized employing housekeeping gene *β-tubulin* (Bm-tub-1). Male worms also showed almost similar results as those of female worms with marginally higher reduction in transcript levels at 24 h [siRNA1 (204-fold, *t*_(100)_ = 39.47, *P* < 0.001), siRNA2 (205-fold, *t*_(100)_ = 39.63, *P* < 0.001), siRNA1 + 2 (262-fold, *t*_(100)_ = 50.75, *P* < 0.001] and at 72 h [siRNA1 (410-fold, *t*_(100)_ = 79.42, *P* < 0.001), siRNA2 (404-fold, *t*_(100)_ = 78.33, *P* < 0.001), siRNA1 + 2 (505-fold, *t*_(100)_ = 97.97, *P* < 0.001)] (Fig. 9a-f).

The observations made at the protein levels of Bm_UGM post-treatment substantiated the qRT-PCR data. There was decline at protein levels at 24 h of treatment which amplified profoundly at 72 hand remained so up to 120 h. The graph has been plotted in terms of band density (Additional file 1: Figure S1).

### In vitro gene silencing caused L3 lethality and impaired in vivo development

**Fig. 10:**
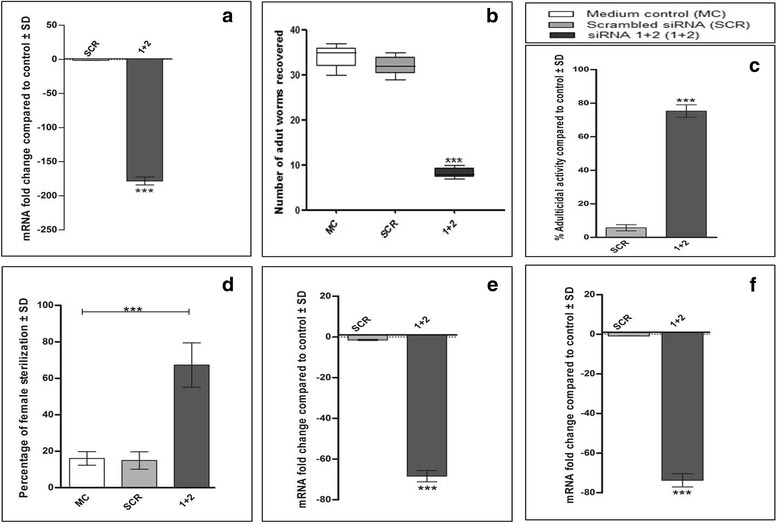
Effect of in vitro gene silencing on L3 and their in vivo development in jirds. In vitro treatment of L3 with siRNA1 + 2 resulted into reduction in *bmugm* gene expression and impaired their in vivo development in jirds. **a** siRNA treatment of L3 resulted in a significant knockdown in *bmugm* gene expression after 48 h of incubation. **b, c** L3 incubated with siRNA1 + 2, negative siRNA and in medium for 48 h were inoculated in the peritoneal cavity of jirds followed by euthanization on 120-day post-inoculation for recovery of adult worms which revealed significantly reduced in vivo adult worms establishment in siRNA1 + 2 group. **d** Significant number of female worms recovered from siRNA-treated group displayed impaired embryogenesis. Percent of sterile females was calculated with respect to total female worms recovered. The fold downregulation in target gene expression in vivo recovered females (**e**) and males (**f**) was also quantified by Real time PCR. ***P < 0.001 (very highly significant)

The in vitro silencing caused by siRNA1 + 2 was marginally superior to each specific siRNA and therefore this combination was used for soaking L3 to investigate the in vitro effects followed by their further development and establishment in the peritoneal cavity of jirds. Of the L3 larvae soaked in siRNA1 + 2 for 24 h, 14.0% were actively motile while majority (~76.5%) became sluggish and a few (9.5%) died. On siRNA treatment for 48 h, only 9.5% remained active and majority of the larvae (~51.2%) died while 39.2% turned sluggish (Table 7) in contrast to actively motile larvae in controls (~1–3% slightly sluggish). There was cut down in the *bmugm* transcript level of treated L3 (sluggish) by ~178-fold over that of scrambled siRNA-treated control worms (1.4-fold) as compared to medium control larvae control (Fig. 10a). The actively motile L3 from cultures at the end of 48 h treatment with target siRNAs, negative control and medium control were inoculated into the peritoneal cavity of 6 week-old male jird (4/group). Jirds euthanized 120 days post-infection revealed drastically reduced (~75.3%) adult worm establishment (*t*-test: *t*_(22)_ = 64.09, *P* < 0.001) (Fig. 10b, c) in addition to sterilization in ~67.3% (*t*-test: *t*_(22)_ = 13.72, *P* < 0.001) of the established live female worms demonstrating impaired embryogenesis (Fig. 10d).

**Table 7:** Effect of siRNA treatment on infective larvae (L3) (mean number ± standard deviation)

L3 condition	MC		SCR		siRNA1 + 2	
	24 h	48 h	24 h	48 h	24 h	48 h
Active (%)	99.0 ± 0.71	96.5 ± 1.41	97.5 ± 0.27	95.7 ± 0.47	14.0 ± 0.71	9.5 ± 0.64
Sluggish (%)	1.0 ± 0.78	3.5 ± 1.42	2.5 ± 0.97	4.2 ± 1.68	76.5 ± 1.97***	39.2 ± 3.94**
Dead (%)	0	0	0	0	9.50 ± 0.50	51.2 ± 2.83***

*Abbreviations*: *MC* medium control; *SCR* negative control (scrambled siRNA)

**P < 0.01; ***P < 0.001

The adult worms recovered from jirds were also quantified by qRT-PCR for *bmugm* gene expression which revealed 68.4–73.7-fold (*t*-test: *t*_(22)_ = 116.1 (female) and *t*_(22)_ = 88.51 (male), *P* < 0.001) reduced transcript levels over control parasites (Fig. 10e, f). No significant reduction was noticed in scrambled siRNA-treated group. The worms recovered from jirds inoculated with target siRNA-treated L3 were weak as compared to healthy worms of medium control and negative control; however, there was no noticeable change in the length of adult parasites from those of control groups (Table 8).

**Table 8:** Effect of silencing on the length (mean ± standard deviation) of male and female worms recovered from jirds infected with in vitro siRNA-treated (*bmugm* specific, negative) and untreated L3

Groups	Worm length (cm)	
	Female worms	Male worms
Medium control	3.7 ± 0.09	1.75 ± 0.06
Scrambled siRNA	3.7 ± 0.08	1.72 ± 0.1
siRNA1 + 2	3.5 ± 0.07**	1.58 ± 0.05*

*P < 0.05; **P < 0.01

## Discussion

Mass drug administration is given annually in a single dose for several years to keep microfilarial levels low to interrupt further transmission. The suboptimal response of filaricides together with the emergence of drug resistance advocates discovery of newer chemical moieties and novel drug targets [24–26]. The reverse genetics approach using gene silencing techniques employing small interfering RNAs is being utilized to study gene associated disorders such as cancer, infectious, respiratory and neurodegenerative diseases. RNAi has emerged as a novel and potent tool that regulates post-transcriptional gene expression and helps in functional analysis of target gene/s in several diseases. This technique in *C. elegans* has greatly helped in excavating the functionality of genes orthologous in *B. malayi.* A full RNAi screen database is available online showing the phenotypic effects caused by silencing of genes (www.rnai.org). RNAi is being progressively used for predicting potential roles of the genes involved in host-parasite interactions and vital processes essential for survival in many parasitic helminths [27] such as *Nippostrongylus brasiliensis* [28], *Ascaris suum* [29, 30], *Haemonchus contortus* [31–33], *Litomosoides sigmodontis* [34], *Onchocerca volvulus* [35, 36], *Trichostrongylus colubriformis* [37] and *Ostertagia ostertagi* [38]. Recently, an in vivo gene silencing study has been also undertaken in *B. malayi* mosquito vector *A. aegypti* [39]. Cathepsin-like enzymes, [40], *β-tubulin* (Bm-tub-1), RNA polymerase II large subunit (Bm-ama-1), mf sheath protein (Bm-shp-1) [41] and cysteine proteases [42] have been functionally analyzed by soaking *B. malayi* worms in siRNA which led to impaired parasite survival and fecundity. Soaking method has been found to be better in parasitic helminths and few essential requirements need to be kept in mind before selecting an appropriate siRNA that should include not only sequence specificity but also most favorable length, concentration, 3’ dinucleotide overhangs along with low G/C content [43–46]. Studies have shown that small size siRNAs in low concentrations are more effective and cause less off-target effects than higher concentrations [47, 48]. We earlier characterized and evaluated the essentiality of few genes of *B. malayi* such as ATPase RNA helicase,trehalose-6-phosphate phosphatase (TPP), independent phosphoglycerate mutase (iPGM) [17, 18, 21] by RNAi using single target gene sequence of siRNA; however, improved silencing effects with minimal chances of off target effects could have been obtained if more than one siRNA were used. In the current investigation, two different siRNA sequences targeting same gene were employed anticipating better *bmugm* gene silencing, a non-*B. malayi* targeted scrambled siRNA (negative control) was simultaneously used throughout the experiment to observe the off target effects, if any. The siRNAs were picked up by parasite within 24 h of soaking and were visualized microscopically till the end of exposure period. In vitro gene knockdown using siRNA1 and siRNA2 individually as well as pooled siRNAs1 + 2 of *bmugm* caused impairment in the viability of exposed worms and tremendously reduced the parasite motility by ~98–99% within 72 h. It was interesting to observe that adverse effects were retained even after 48 h of replacing the medium with fresh siRNA free medium. In addition to worm motility or viability, the release of mf by gravid females and their motility was also abridged up to large extent. Gene silencing had tremendous impact on the embryogenesis. More and more early eggs were visible with decrease in advanced developing stages and degeneration of intrauterine stages suggesting phenotypic deformities and development arrest. Real time data revealed knockdown in *bmugm* expression which amplified further with the exposure duration and persisted even after removal of siRNA. The pooled siRNA effects were ~100-fold more than in presence of siRNA 1or 2 individually. Both sexes of worms were affected in almost similar fashion. In view of better gene silencing effects of two siRNAs, this combination was exploited in case of infective larvae where deleterious effects were conspicuous making them sluggish, and/or immotile. The treated but actively motile larvae on transfer to healthy susceptible host, jirds demonstrated their highly reduced transformation in to adult worms and recovered female worms had embryostatic effects. Although the size of worms was within limits, they were comparatively much thinner than those recovered from animals receiving untreated larvae. In jirds, L3 takes around 3 months to develop into sexually mature adult parasites; however, the worms developed from siRNA-treated larvae demonstrated ~2.6 times lower mRNA expression of target gene than detected in L3s soaked for 48 h in siRNA medium. On the contrary, gene expression in adults established from L3 soaked in scrambled siRNA remained unchanged. These findings suggest that *bmugm* gene knockdown effect was partly reversible although lasted longer even when the larvae grew and transformed into mature adult parasites in jirds.

In *C. elegans,* mutation in *ugm* gene has been shown to culminate in a series of phenotypic defects like traction impairment, superficial blisters, body constrictions, larval lethality, hyper-permeability, drug sensitivity, cuticle fragility and loss, depletion or masking of outer cuticle components [9, 49–53] suggesing the essentiality of this gene. The current findings demonstrated impaired adult and larval motility, viability and lethality, embryostasis, and other phenotypic alterations as a result of knockdown in *bmugm* gene expression.

It may therefore be inferred that *bmugm* is an essential gene in lymphatic filarial parasite, *B. malayi* which is imperative for their development and survival and thus shows its worth to be picked up as a vital gene target for chemotherapeutic intervention.

## Conclusions

The discovery of alternative chemical moieties with differing mode of action, validated antifilarial drug target and a vaccine to combat filariasis is urgently needed. siRNA mediated gene silencing or compound inhibitor studies are the tools to analyze function of a gene. UGM is widely distributed among nematodes; however, its complete absence in mammals renders it an interesting putative anthelmintic drug target. The drug targets in helminths including filariids are almost non-existent. RNAi experiments using two *B. malayi ugm-*specific siRNAs in combination carried out in current study revealed the essentiality of *bmugm* in filarial metabolism. The studies were undertaken both in vitro and in vivo which caused profound adverse effects on parasite survival and viability correlating with profound knockdown of the target gene expression. The gene silencing effects were long-lasting; however, partial reversal in reduced gene expression was noticed in established adult parasites after 3 months of transfer of treated larvae in to jirds. The gene knockdown was specific, no off-target effects were noticed and two specific siRNA targeting two different prioritized gene sequences in combination showed better gene knockdown than single siRNA. The current findings thus validate *bmugm* as a potential anti-filarial drug target. Further crystallization and docking studies would facilitate rationale drug designing for identifying the inhibitors against this target.

## Additional file

Additional file 1: Figure S1. The graph bars depict percent relative intensity of bands on western blot in comparison to medium control at different time points of exposure (24, 48, 72 h) and after exposure (96, 120 h) as quantified by imageJ version 1.47 software and the blot bands have been shown below each corresponding bar. Considering the medium control intensity to be 100% at each time points the relative intensities of treated ones have been evaluated. *P*-value < 0.05 was considered significant (*), *P* < 0.01 as highly significant (**) and *P* < 0.001 as very highly significant (***). (TIF 4078 kb)

## Abbreviations

Bm_UGM: *B. malayi* UDP-Galactopyranose mutase protein
*bmugm*: *B. malayi* UDP-Galactopyranose mutase gene
siRNA: small interfering ribonucleic acids
UDP-Gal*f*: UDP-Galactofuranose
UDP-Gal*p*: UDP-Galactopyranose
