# Supplementary figures and images for: RNA interference mediated knockdown of Brugia malayi UDP-Galactopyranose mutase severely affects parasite viability, embryogenesis and in vivo development of infective larvae
Source: Parasit Vectors. 2017 Jan 19;10:34. doi: 10.1186/s13071-017-1967-1 (PMC5244609; doi:10.1186/s13071-017-1967-1)

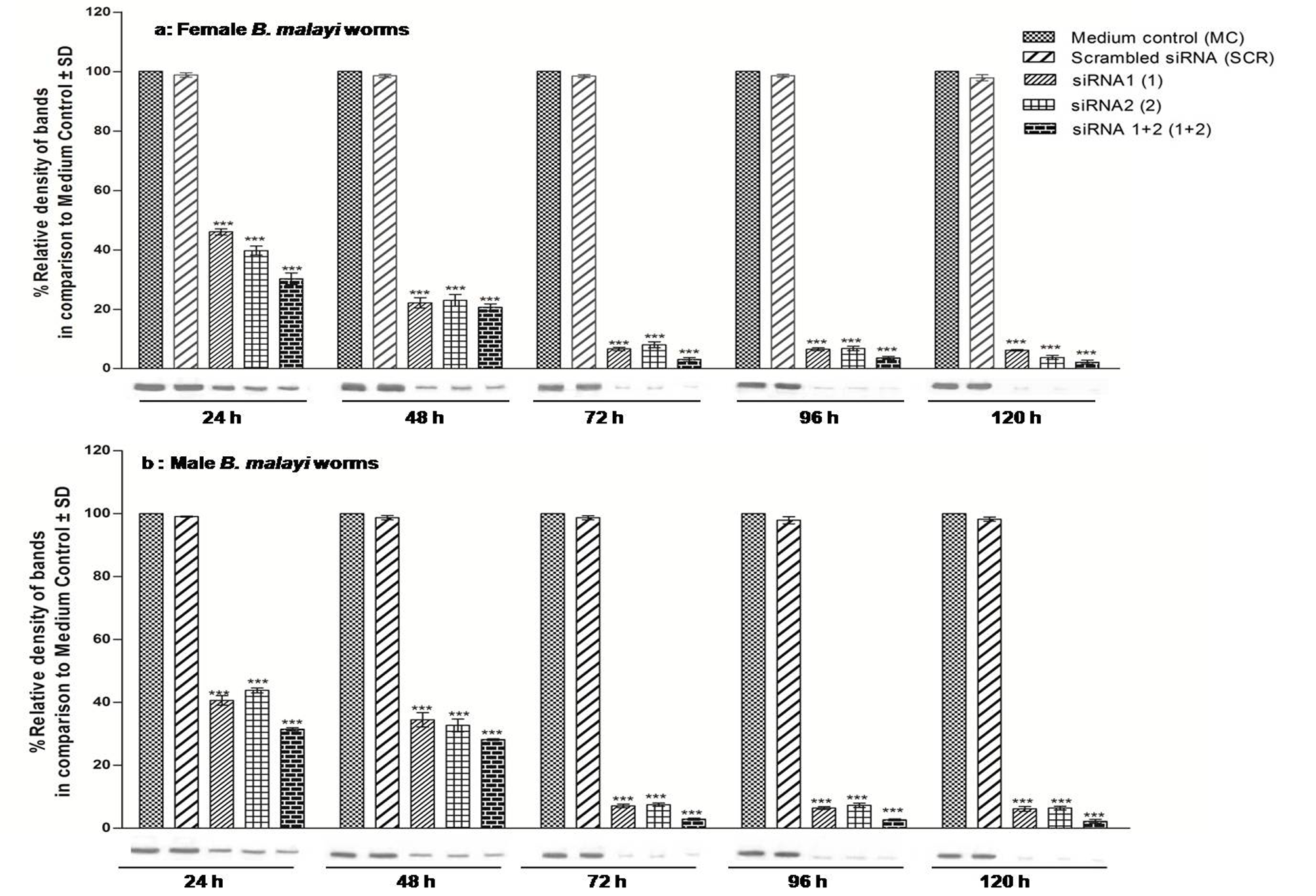

Supplement: Additional file 1: Figure S1. — The graph bars depict percent relative intensity of bands on western blot in comparison to medium control at different time points of exposure (24, 48, 72 h) and after exposure (96, 120 h) as quantified by imageJ version 1.47 software and the blot bands have been shown below each corresponding bar. Considering the medium control intensity to be 100% at each time points the relative intensities of treated ones have been evaluated. P-value < 0.05 was considered significant (*), P < 0.01 as highly significant (**) and P < 0.001 as very highly significant (***). (TIF 4078 kb) [file 13071_2017_1967_MOESM1_ESM.tif]
